# Herpes simplex virus infection and Alzheimer’s disease

**DOI:** 10.3389/fnagi.2026.1858017

**Published:** 2026-08-20

**Authors:** Ji-Yeun Hur

**Affiliations:** Department of Biological Sciences, Ajou University, Suwon, Republic of Korea

**Keywords:** Alzheimer’s disease, amyloid beta, antimicrobial peptide, herpes simplex virus, neuroinflammation, neurotropic virus, tau, viral infection

## Abstract

Over 95% of Alzheimer’s disease (AD) is caused by a complex mixture of genetic and environmental factors. This is called sporadic AD or late-onset AD, and much of its pathological mechanisms remain unclear. There is growing evidence indicating that the viral infection, such as herpes simplex virus type 1 (HSV-1), triggers and worsens the AD progression in combination with the APOE4 genotype in AD patients. The innate immunity of host cells produces proinflammatory cytokines, which further drive neuroinflammation in the brain. Concurrently, Aβ and phosphorylated tau could entrap foreign pathogens such as HSV-1 according to the “antimicrobial protection hypothesis of AD,” and accelerate the progression of AD. In this review, growing evidence ranging from HSV-1 infection to the AD progression via the accumulation of Aβ and tau, and neuroinflammation is explored.

## 1 Introduction

Alzheimer’s disease (AD) is the most common form of dementia (Terry and Davies, 1980). There are an estimated 7.4 million AD populations in the USA, and the projection is estimated to grow to 13.8 million by 2060 (Alzheimer's Association, 2026). AD patients show cognitive symptoms, including memory impairment, due to neuronal cell death and synapse loss in the brain (Masliah et al., 2001; Shankar and Walsh, 2009). These are caused by the aggregation of toxic proteins and neuroinflammation in the brain. There are two major pathological hallmarks in the brain of AD patients: senile plaques, which are the extracellular aggregation of amyloid β (Aβ), and neurofibrillary tangles, which are the intracellular aggregation of hyperphosphorylated tau protein in neurons (St George-Hyslop, 2000; Perl, 2010). AD progresses from preclinical AD to mild cognitive impairment (MCI), mild, moderate, and severe AD (Tahami Monfared et al., 2022). Aβ42 levels in the cerebrospinal fluid (CSF) decrease while Aβ deposition increases in AD brains, already about 30 years prior to the onset of symptoms. These changes are followed by an increase in CSF tau levels, brain structural changes, and eventually, clinical symptoms such as memory decline over time (Bateman et al., 2012).

Aβ is produced by sequential cleavages of β-secretase and γ-secretase of the amyloid precursor protein (APP), and this is called the amyloidogenic pathway (Selkoe, 2001). γ-Secretase is a transmembrane protein complex consisting of at least four mandatory protein subunits, presenilin 1 (PS1), nicastrin, anterior pharynx-defective 1 (Aph-1), and presenilin enhancer 2 (Pen-2) (Kimberly et al., 2003). According to the amyloid hypothesis in AD, the imbalance between Aβ production and Aβ clearance leads to the aggregation of Aβ, followed by microglial and astrocytic activation (neuroinflammation), neuritic injury, tangle formation, cell death, and dementia (Hardy and Selkoe, 2002). Resulted Aβ species from the amyloidogenic pathway vary from 37 to 43 amino acids long, and Aβ42 is the major component of amyloid plaques (Iwatsubo et al., 1994; Roher et al., 1993). The major Aβ species produced are Aβ40 and Aβ42, and there is a ratio of about one to nine for Aβ42:Aβ40 (Iwatsubo et al., 1994). Only two amino acid differences make Aβ42 as more prone to aggregate and toxic species than Aβ40 (Iwatsubo et al., 1994). Aβ polymerizes from monomers, dimers/trimers, oligomers, protofibrils, fibrils, and plaques in AD (Hardy and Selkoe, 2002). Tau protein binds to tubulin and stabilizes microtubules, one of the cytoskeleton proteins in cells (Iqbal et al., 2005; Barbier et al., 2019). Hyperphosphorylated tau is dissociated from microtubules and becomes aggregated as neurofibrillary tangles (Iqbal et al., 2005). There are six isoforms of tau in the human brain, and 85 phosphorylation sites were reported (Iqbal et al., 2005; Parra Bravo et al., 2024; Donison et al., 2025).

There are two types of AD: familial AD [FAD, also referred to as early-onset AD (EOAD)] and sporadic AD [SAD, also referred to as late-onset AD (LOAD)]. FAD affects about 1–2% of AD patients, and familial mutations on *APP*, *PSEN1* (PS1 gene), and *PSEN2* (an isoform of PS1) of the APP processing increase the ratio of Aβ42/Aβ40 and/or total Aβ production (Holtzman et al., 2011; Bettens et al., 2010). The majority of AD patients are SAD patients, who have a complex mixture of genetic and environmental factors, and SAD has lowered clearance of Aβ (Bettens et al., 2010). The biggest risk factor for AD is aging (Querfurth and LaFerla, 2010). Another major genetic risk factor is apolipoprotein E (APOE). The APOE gene codes for a protein involved in lipid transport. There are three alleles in APOE: APOE2, APOE3, and APOE4. There are amino residue substitutions from cysteine to arginine in APOE alleles: Cys112/Cys158 for APOE2, Cys112/Arg158 for APOE3, and Arg112/Arg158 for APOE4 (Poirier, 2005). APOE3 is the most common allele in the population. APOE4 is a risk factor for SAD, while APOE2 has a protective effect against AD (Poirier, 2000). In addition, as the copy number of the APOE4 allele increases, the age of onset of AD decreases in a gene-dose-dependent manner (Corder et al., 1993). The isoforms of APOE affect Aβ clearance from the brain differently; APOE4 has the least clearance of Aβ (Deane et al., 2008). APP transcription and Aβ levels also increase in the order of APOE4 > APOE3 > APOE2, indicating APOE4 could activate neuronal Aβ synthesis (Huang et al., 2017). The meta-analysis shows that approximately 40% of SAD patients have at least one copy of the APOE4 allele (Farrer et al., 1997).

In recent years, genome-wide association studies (GWAS) have shown that immune response related genes such as triggering receptor expressed on myeloid cells 2 (*TREM2*) and *CD33* are implicated in SAD (Griciuc and Tanzi, 2021). TREM2 is expressed in immune cells, including microglia, the brain resident immune cells, whose major function is to phagocytose Aβ. *TREM2 R47H* variant induces microglial dysfunction and reduces phagocytosis of Aβ, therefore it is an AD risk gene (Griciuc and Tanzi, 2021). Less compact amyloid plaques together with more severe neuritic tau hyperphosphorylation were shown by immunofluorescence imaging in the brain of SAD with *TREM2 R47H* variant due to impaired Aβ phagocytosis (Yuan et al., 2016). These variants are either rare or common in the population, and they may have a small, moderate, or large effect on AD risk (Karch and Goate, 2015). Humans have a long life span, and every individual harbors a different genetic makeup and goes through different environmental factors in their life. Therefore, SAD is still unclear. Recent developments in AD immunotherapy, such as aducanumab (Aduhelm^®^), lecanemab (Leqembi^®^), and donanemab (Kisunla^®^), target different forms of Aβ: 3–7 amino acids of Aβ42 peptide binding fibrillar aggregates, Aβ1–16 binding soluble Aβ aggregates such as oligomers and protofibrils, and N-terminal pyroglutamate Aβ binding Aβ plaques, respectively (Cummings et al., 2024; Arndt et al., 2018; Strohl, 2024; Soderberg et al., 2023).

These clinical trials with anti-Aβ monoclonal antibodies showed that APOE4 allele carriers showed increased risk to develop amyloid-related imaging abnormalities (ARIA), including oedema (ARIA-E) and microhaemorrhages (ARIA-H) (Cummings et al., 2024; Hampel et al., 2023). Thus, lecanemab is used in APOE4 non-carriers or heterozygotes with confirmed amyloid pathology (Perry et al., 2026). Overall, AD can be heterogeneous based on APOE genotypes, and the effect of each treatment should be considered in combination with genetic factors. In addition, APOE4 alone is not sufficient to induce AD; therefore, other genetic or environmental factors might play a role in combination (Itzhaki et al., 1997).

## 2 HSV-1 infection and AD

There is growing evidence indicating that chronic bacterial and viral infection poses a risk for developing neurodegenerative disorders. Various studies suggest that the possible implication of herpes simplex virus type 1 (HSV-1) and *Chlamydia pneumoniae* in AD, influenza in Parkinson’s disease, enteroviruses and human herpesviruses (HHV) in amyotrophic lateral sclerosis, and HSV, Epstein Barr Virus (EBV), varicella zoster virus (VZV), cytomegalovirus (CMV), human herpesvirus 6 (HHV-6), and human herpesvirus 7 (HHV-7) in multiple sclerosis (De Chiara et al., 2012; Ingvarsson et al., 2026). Thus, infections could be one of the environmental factors for the etiology of SAD.

**Figure 1:**
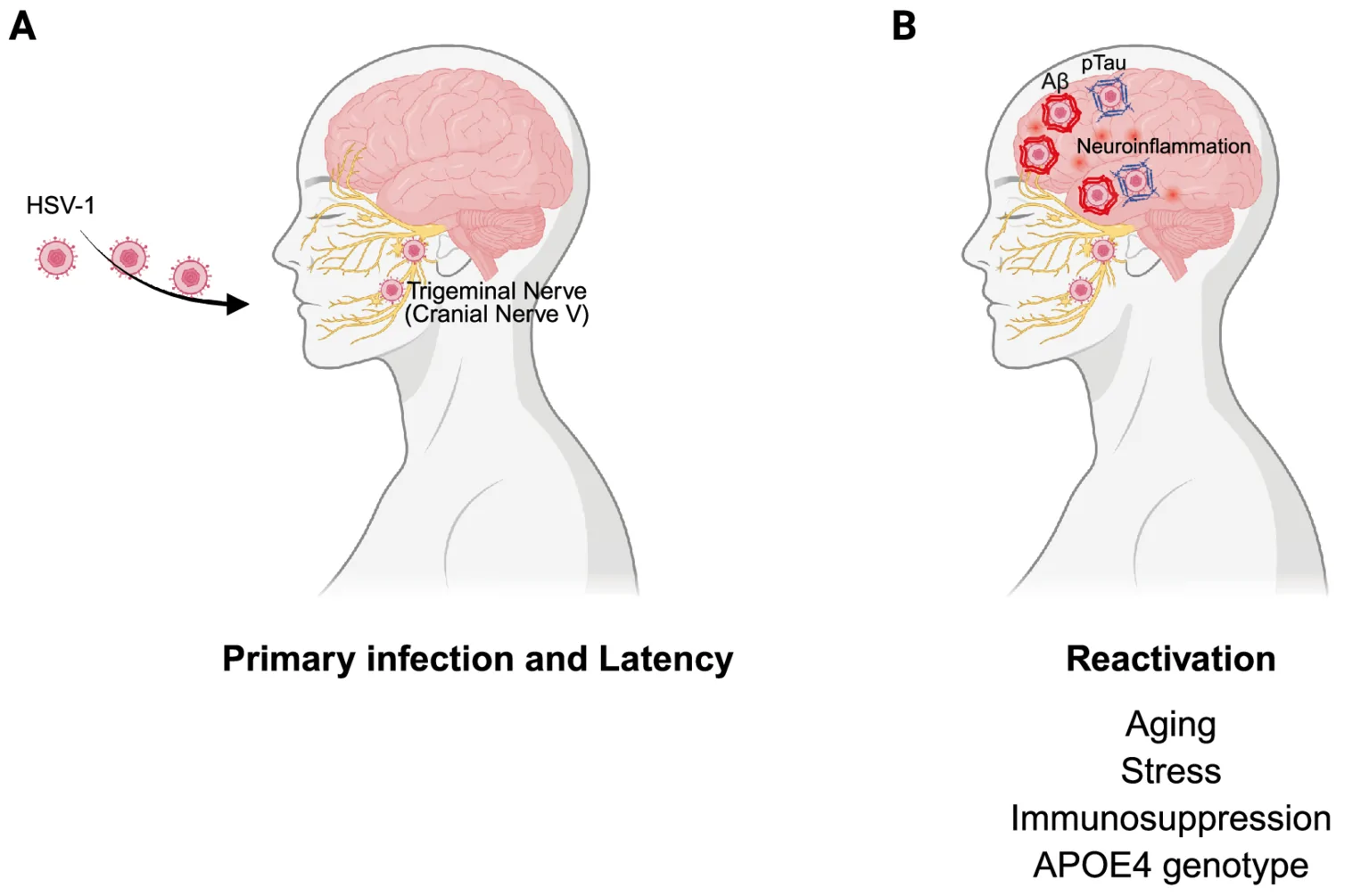
HSV-1 infection in the brain and AD. **(A)** HSV-1 infects via skin-to-skin contact, and possible viral entry routes to the CNS include via olfactory bulbs, and HSV-1 moves via axonal transport to the trigeminal nerve (cranial nerve V). **(B)** HSV-1 can be reactivated under various conditions such as aging, stress, immunosuppression, etc. The “antimicrobial protection hypothesis of AD” suggests that Aβ and phosphorylated tau (pTau) could work as antimicrobial peptides (AMPs) and entrap HSV-1, preventing it from spreading. Concurrently, the process of aggregation of Aβ and hyperphosphorylated tau could lead to AD progression. HSV-1 reactivation, as well as the aggregation of Aβ and hyperphosphorylated tau enhance neuroinflammation further by inducing proinflammatory cytokines. Additionally, the combination of HSV-1 infection and a genetic makeup such as a copy number of the APOE4 allele increase a risk for AD. Overall, these altogether contribute to the progression of AD.

Herpesviridae (herpesviruses) is a family of DNA viruses including α-herpesviruses (HSV-1, HSV type 2 (HSV-2), and VZV), β-herpesviruses (CMV, HHV-6, and HHV-7), and γ-herpesviruses (EBV and human herpesvirus 8) (McGeoch et al., 1995; Marshall et al., 2024). There are two types of HSV: HSV-1 infection causes oral herpes and cold sores, while HSV-2 is contracted through sexual contact. Both HSV-1 and HSV-2 can cause genital herpes (Ashley-Morrow et al., 2004). The prevalence of HSV-1 infection in the global population under 50 years old is estimated at 63.6% for oral HSV-1, 5.2% for genital HSV-1, and 66.6% for HSV-1 at any site (James et al., 2016). The estimated global prevalence of HSV-2 among individuals aged 15–49 years old is 13.2% (James et al., 2016). HSV-1 infection often occurs in childhood, and the infection stays lifelong with periodic reactivation (Petti and Lodi, 2019). Most individuals with HSV-1 infection remain asymptomatic but are still infectious with shedding viruses in tears, nasal and oral mucosa, and oral cavity (Petti and Lodi, 2019; Ramchandani et al., 2016). HSV-1 is a neurotropic virus that can infect the central nervous system (CNS) via retrograde axonal transport after primary infection (Sait et al., 2021). HSV-1 can stay dormant in the trigeminal nerves (cranial nerve V) (referred to as HSV latency) and can be reactivated later in life (referred to as HSV reactivation) under aging, stress, and a weakened immune system, such as immunosuppression (Sait et al., 2021) (Figure 1).

There is growing evidence that HSV-1 infection might be associated with AD. The “infectious hypothesis” suggests that infections might lead to AD (Seaks and Wilcock, 2020; Sochocka et al., 2017). To find the footprints of pathogens, researchers have found a virus in the brain (Dobson and Itzhaki, 1999). Itzhaki et al. found the latent form of HSV-1 as the sequence of HSV-1 DNA (HSV-1 thymidine kinase gene) in the postmortem brains of the elderly and AD by polymerase chain reaction (PCR) (Itzhaki et al., 1997; Jamieson et al., 1991). The brain regions (temporal and frontal lobes) from 8 AD cases and 6 normal controls, including both males and females, were analyzed, and PCR results show that the sequence of the HSV-1 thymidine kinase gene is present in all postmortem elderly human brain samples, including both AD and normal controls (Jamieson et al., 1991). Additionally, Itzhaki et al. carried out PCR amplification to detect the HSV-1 thymidine kinase gene and the APOE gene from hosts using DNA extracted from brain regions (temporal and frontal lobes) of 46 AD patients and 44 elderly controls (Itzhaki et al., 1997). The results show that the frequency of the APOE4 allele is significantly higher in the HSV-1-positive AD group, compared to the HSV-1-negative control group [odds ratio (OR) = 16.8] in brain samples (Itzhaki et al., 1997). It was also found that the frequency of the APOE4 allele is significantly higher in recurrent cold-sore sufferers (36.3%) compared to non-sufferers (9.1%) using serum samples (OR = 5.69), and the authors conclude that reactivation of HSV-1 is strongly affected in APOE4 allele carriers (Itzhaki et al., 1997). Although HSV-1 infection in APOE4 allele carriers is associated with an AD risk increase, a single risk factor such as APOE4 or HSV-1 infection alone, or both combinations, is not sufficient to induce SAD (Itzhaki et al., 1997). Interestingly, the authors stated that APOE4 is also a risk factor for cold sores (Itzhaki et al., 1997). Wozniak et al. reported that the DNA of HSV-1 was found in the amyloid plaques of AD and control brains (Wozniak et al., 2009). In *situ* PCR was used to detect HSV-1 DNA in postmortem human brains (frontal and temporal cortices) from 6 AD patients (77 years old as a mean age, 58% of APOE4 allele frequency) and 5 controls (79 years old as a mean age, 0% of APOE4 allele frequency), followed by thioflavin S staining to detect *β*-sheet structures in amyloid plaques, and co-localization of HSV-1 DNA and amyloid plaques was found both in AD and normal control brains (Wozniak et al., 2009). The authors suggest that the APOE4 allele in combination with HSV-1 could play a major role in determining AD progression, noting that all control cases are negative for the APOE4 allele (Wozniak et al., 2009). Letenneur et al. assessed titers of serum immunoglobulin G (IgG) (indicating past infections) and IgM (indicating primary infection or reactivation) for HSV in a cohort study of normal and pathological cerebral aging in France, where 512 non-demented elderly subjects were followed for 14 years (Letenneur et al., 2008). A hazard ratio (HR) of anti-HSV IgG status in developing AD was not found to be significant between IgG-positive and IgG-negative groups, while anti-HSV IgM status was significantly associated with increased risk of developing AD (HR = 2.55) (Letenneur et al., 2008). In this study, anti-HSV Ig titers include both HSV-1 and HSV-2 (Letenneur et al., 2008). Although IgM positivity suggests recent HSV reactivation, the authors stated that there was no information available for possible HSV reactivation (Letenneur et al., 2008).

Other herpesviruses also could remain latent (Carbone et al., 2014). HHV-6A and HHV-7 are reported to be associated with AD using SAD-associated virome combining from genomic, transcriptomic, proteomic, clinical, and neuropathological data of postmortem human brains (Readhead et al., 2018), while no association was reported between HSV-1 (Hemling et al., 2003; Westman et al., 2017; Aiello et al., 2006), HHV-6 (Hemling et al., 2003; Allnutt et al., 2020; Agostini et al., 2016), VZV (Hemling et al., 2003; Westman et al., 2017), and CMV (Carbone et al., 2014; Westman et al., 2017) and AD. PCR results for the HSV-1 glycoprotein B (gB) gene in the brains of 34 AD patients and 40 controls in the Finnish population show that only 2.9% of AD patients were positive for HSV-1 DNA (Hemling et al., 2003). The positivity for HHV-6 DNA and VZV DNA were 88.2 and 26.5% in AD samples, and there was no difference between AD patients and controls (Hemling et al., 2003). In a study with plasma samples from 50 AD patients and 52 non-demented controls, a significantly lower HHV-6 IgG reactivity was reported in AD (Westman et al., 2017). Apart from HSV-1, CMV is also prevalent in the elderly population (Aiello et al., 2006). In a community-dwelling elderly population, a study using serum samples (*N* = 1,204) from the Sacramento Area Latino Study on Aging (SALSA) suggested that increased CMV IgG levels were associated with cognitive decline over a 4-year period, and there was no correlation between HSV-1 IgG levels and cognitive decline (Aiello et al., 2006). Carbone et al. reported that EBV and HHV-6, but not CMV, might be environmental risk factors for cognitive decline and AD progression (Carbone et al., 2014). Using peripheral blood leukocytes from AD patients and controls, the positivity for EBV DNA was reported to be 45.5% in AD and 31.1% in controls (OR = 1.843), and 23.1% in AD and 4.4% in controls (OR = 6.5) for HHV-6 DNA (Carbone et al., 2014). Using DNA from the frontal cortices of AD patients, positivity for EBV and HHV-6 was reported to be 5.9% and 17.3% in AD (Carbone et al., 2014). Although only 5.9% of AD samples were positive for the EBV DNA, these samples all came from APOE4 carriers. There was no difference between APOE4 carriers and non-carriers for the HHV-6-positive samples (Carbone et al., 2014). IgG levels of CMV and EBV in plasma samples showed no difference between controls and AD (Carbone et al., 2014). Interestingly, these IgG titers were significantly increased in controls who developed AD later, compared to controls who remained cognitively intact (Carbone et al., 2014).

Bacterial infection by *Porphyromonas gingivalis* (*P. gingivalis*) in gum disease has also been linked to AD (Beydoun et al., 2020). A retrospective cohort study using the National Health and Nutrition Examination Survey III in the US, which measured IgG titers in serum for humoral immune response against periodontal bacteria, showed that *P. gingivalis* IgG titers were associated with increased risk for AD in women (Beydoun et al., 2020). Fungal DNA was also found in the CNS samples of AD patients (Pisa et al., 2015). In addition, HSV-1 can cause another CNS infection, herpes simplex encephalitis (HSE), which causes severe cognitive sequelae such as memory loss and cognitive impairment (Campos et al., 2021). While encephalitis is caused by many factors, the most common cause is neurotropic viruses, and HSV-1 accounts for 50–70% of viral cases (Tyler, 2018). Thus, this suggests that viral infection may be a potential etiology and environmental factor for SAD, and it opens new therapeutic avenues, focusing on antiviral treatments and vaccinations as potential strategies to prevent or slow the progression of the disease.

## 3 Association studies between HSV-1 infection and AD risk

Growing evidence indicates that HSV-1 infection could lead to AD pathogenesis as well as neurodegeneration. A nationwide and population-based study in Taiwan using National Health Insurance Research Database reported that the HSV cohort including newly diagnosed individuals over 50 years old with HSV-1 or HSV-2 infection were associated with an increased risk of developing dementia by a 2.56-fold (adjusted hazard ratio (HR) = 2.564) compared to age matched control cohort, and anti-herpetic medications were associated with a lowered risk of dementia including AD, vascular dementia, and other dementia in the treated HSV cohort by 90.8% (adjusted HR = 0.092), compared to non-treated HSV group (Tzeng et al., 2018). In addition, there was an increased risk of AD only in the HSV-1 infection group, while the risk of vascular dementia and other dementias was increased by both HSV-1 and HSV-2 infections (Tzeng et al., 2018). However, authors also stated the limitations of their retrospective database study, which lacks certain data, such as HSV serology and APOE4 genotype. Additionally, authors noted that most dementia cases in the study are from other types rather than AD, which is the most common type of dementia (Tzeng et al., 2018). Similarly, Liu et al. investigated the association between HSV-1 and AD in a retrospective and matched case–control study using a large administrative claims database from the USA (Liu et al., 2025). AD cases are matched to controls with a 1:1 ratio on covariates such as age, sex, region, database entry year, and healthcare visit numbers (Liu et al., 2025). Out of 344,628 AD patients aged over 50 years old, 1,507 (0.44%) had a history of HSV-1 diagnosis compared to 823 (0.24%) in controls. A diagnosis of HSV-1 was associated with AD, with an adjusted OR of 1.80, compared to no diagnosis of HSV-1. Stratified analysis, dividing into age groups, showed that this association was stronger in older age groups of over 75 years old with adjusted OR 2.10, compared to subgroups of 50–70 years (adjusted OR = 1.14) and 71–74 years (adjusted OR = 1.51) (Liu et al., 2025). The usage of anti-herpetic medications after infection was shown to be associated with reduced risk of AD in AD cases with HSV-1 diagnosis (adjusted HR = 0.83) (Liu et al., 2025). It is worth mentioning that a number of AD cases with a history of other herpesviruses, such as HSV-2 (0.17%) and VZV (4.80%), were also significantly higher compared to controls (0.11% with HSV-2 and 3.25% with VZV). On the other hand, a number of individuals with a history of CMV showed no difference between the AD and control groups (Liu et al., 2025). The authors stated that potential limitations of this study could be representing a small fraction of the real population with HSV-1 infection, because asymptomatic cases often do not seek medical care (Liu et al., 2025). Similar results reported that the herpesvirus (HSV or VZV) infection without antiviral treatment increased the risk of dementia (adjusted HR = 1.50) and antiviral treatment was associated with a reduced risk of dementia (adjusted HR = 0.89) based on a registry-based cohort study in Sweden (Lopatko Lindman et al., 2021).

The association between vaccination or treatment and the reduced AD risk has been investigated. Large-scale electronic health record data in Wales indicate that shingles vaccination (live-attenuated herpes zoster vaccination) decreased the risk of dementia development (Eyting et al., 2025). Interestingly, this protective effect was stronger in women than in men (Eyting et al., 2025). Women have a higher risk of AD than men (Fratiglioni et al., 1997), and further investigation on the correlation between gender and potential therapeutic effects on lowering dementia risk is needed. Whether general antiviral therapy could lower AD or dementia risk would be interesting to investigate in the population. Indeed, the first clinical trial, the VALAD (Valacyclovir Treatment of Alzheimer’s Disease) randomized clinical trial, was conducted to test the antiviral drug valacyclovir for HSV infection, whether to slow AD progression (Devanand et al., 2026). Valacyclovir is a prodrug of acyclovir. The Phase 2 clinical trial with valacyclovir at 4 g/d for 78 weeks in the treatment group of early symptomatic AD (probable AD, MCI with positive AD biomarkers) and seropositivity (IgG or IgM) for HSV-1 or HSV-2 was found to be associated with cognitive worsening compared to the placebo group (Devanand et al., 2026). A valacyclovir treatment group showed a least-squares mean (LSM) change in Alzheimer’s disease Assessment Scale Cognitive (ADAS-Cognitive) of 10.86 compared to 6.92 in the placebo group, and the difference between two groups was 3.93, indicating greater cognitive worsening in the valacyclovir treatment group (Devanand et al., 2026). ^18^F-MK-6240 tau PET standardized uptake value ratio (SUVR) results also showed 0.07 in the treatment group versus -0.04 (Devanand et al., 2026). Higher scores indicate higher levels of amyloid or tau by PET (Devanand et al., 2026). Additionally, the covariate of APOE4 genotype was found to be not significant in this study (Devanand et al., 2026). The authors conclude that valacyclovir did not show efficacy and is not recommended to treat early symptomatic AD with HSV seropositivity (Devanand et al., 2026). The authors also stated that one of the limitations of the study might include a small sample size (60 patients receiving valacyclovir and another 60 receiving a placebo) and that the effect of long-term antiviral treatment has not been studied yet compared to this medium-term treatment with valacyclovir for patients (Devanand et al., 2026). For bacterial infection, the Spring trial investigates an oral drug LHP588 (a next-generation gingipain inhibitor) against the bacteria *P. gingivalis*-positive AD to slow AD progression. The Phase 2 study is ongoing.

## 4 Possible mechanisms between HSV-1 infection and AD pathogenesis

Since SAD is a multifactorial disease, most of its etiologies remain unclear. Whether the relationship between HSV-1 infection and the pathogenesis of AD is due to correlation or causation has not been clear, and mechanistic studies often remain limited. In recent years, the “antimicrobial protection hypothesis of AD” has been suggested as a new physiological function of Aβ in AD. Antimicrobial peptides (AMPs) are known to defend against bacterial, fungal, and viral infections by entrapping microbes (Kumar et al., 2016; Moir et al., 2018; Soscia et al., 2010). Therefore, microbes can no longer spread into neighboring cells. Immunostaining images showed that the HSV-1 infection in the brain of 5xFAD transgenic AD female mice at the age of 5–6 weeks increased the co-localization of HSV-1 and Aβ compared to uninfected 5xFAD mice (Eimer et al., 2018). This result suggests that Aβ might entrap HSV-1 and work as an AMP upon viral infection (Kumar et al., 2016; Moir et al., 2018). Aβ and AMP share similar features: (1) < 50 amino acid length, and (2) properties of oligomerization and fibrillization (Soscia et al., 2010). Recently, Eimer et al. reported that phosphorylated tau (pTau) was formed as a result of HSV-1 infection (Eimer et al., 2026). It was suggested that pTau is also an AMP, and hyperphosphorylated tau was formed as an antiviral response (Eimer et al., 2026). Therefore, a “antimicrobial protection hypothesis of AD” suggests that senile plaque and neurofibrillary tangle formations result from antimicrobial activities of Aβ and pTau, which serve as host defense proteins in the brain as the innate immunity (Eimer et al., 2026). This brain’s innate immunity response can also paradoxically aggregate Aβ and pTau further (Eimer et al., 2018; Eimer et al., 2026). Thus, viral infection associated with the accumulation of Aβ and pTau could pose a risk for AD development and progression.

HSV-1 infects the CNS via retrograde axonal transport, where microtubules are stabilized by tau, and intracellular pathogens take advantage of these microtubule networks for infection (Eimer et al., 2026). HSV is shown to interact with APP during fast anterograde transport (Satpute-Krishnan et al., 2003). The C-terminus of APP recruits kinesin, which moves HSV-containing vesicles along microtubules to the synapse (Satpute-Krishnan et al., 2003). In addition, cofilin-1, which reorganizes the actin cytoskeleton of neuronal cells, increases at the early stage of HSV-1 infection (Wang et al., 2020; Xiang et al., 2012). Postmortem AD human brain sections were stained with rods and aggregates of cofilin-1, which were found to correlate with tau pathology (Rahman et al., 2014). Cofilin-1 also co-labeled with Iba1-positive microglia (Rahman et al., 2014). Although it has not been shown with direct evidence connecting HSV-1 infection to AD pathogenesis via cofilin-1, Wang et al. proposed that dysregulation of cofilin-1 might be a missing link (Wang et al., 2020).

Our innate immune system protects host cells from viral infection. Once viral pathogens cross intrinsic barriers such as skin, mucus, and saliva, the host’s innate immunity is the next line of defense (De Chiara et al., 2012; Cheng et al., 2022). Viral DNA of HSV-1 is recognized by pattern recognition receptors (PRRs), which can induce a cascade of signaling events and gene expression of type I interferon (IFN) and proinflammatory cytokines (Yan and Chen, 2012). These IFNs bind to IFN receptors in infected host cells or nearby host cells to stimulate the IFN signaling pathway and induce gene expression of antiviral factors, IFN-stimulated genes (ISGs) (Miller et al., 2016). A study modeling HSE in mice showed that microglia are the main source of type I IFN expression after sensing double-stranded DNA (dsDNA) of HSV-1 in the cytoplasm via the cyclic GMP-AMP synthase (cGAS)-stimulator of interferon genes (STING) pathway in the CNS (Reinert et al., 2016). Type I IFN is required for antiviral infection in the brain, but excessive type I IFN levels could lead to disease pathologies (Reinert et al., 2021). The brain-resident immune cells, microglia, not only surveil their surroundings to detect unwanted foreign pathogens or cell debris, but also regulate phagocytosis in the brain (Telikani et al., 2022). Resting microglia can shift to disease-associated microglia (DAM), which downregulate the transcriptional signature unique to microglia and upregulate interferon response genes, lysosomal genes, etc. (Song and Colonna, 2018). In neurodegeneration, such as AD, persistent production of aberrant Aβ and tau dysregulates microglial phagocytic function and increases proinflammatory response by microglia (Hickman et al., 2018). Neurons, which conduct synaptic transmission, are non-renewable cells and vulnerable to insult (De Chiara et al., 2012; Telikani et al., 2022). Neuroinflammation triggered by glial cells, including microglia, can further reinforce a disease progression in AD (Colonna and Butovsky, 2017). It is suggested that repeated reactivation of ‘mild’ HSV-1 infection could damage neurons (Marcocci et al., 2020), and aged neurons are more susceptible to damage (Barbier et al., 2019; De Chiara et al., 2012). Infection of HSV-1-green fluorescent protein (GFP) in the hippocampus of 5xFAD mice resulted in increased Aβ deposition, increased microglial phagocytosis for HSV-1-GFP-positive neurons instead of Aβ, and cognitive decline in mice (Wang et al., 2024). NOD-like receptors (NLRs), such as the NLRP3 (NOD-, LRR- and pyrin domain-containing 3) inflammasome, are one of the PRRs expressed in microglia (Colonna and Butovsky, 2017; Prinz and Priller, 2014). HSV-1 infection in 5xFAD mice was also reported to activate NLRP3 inflammasome in microglia, and blocking the NLRP3 inflammasome by a selective small-molecular inhibitor (MCC950 sodium) reduced the number of amyloid plaques in the brains of HSV-1 infected 5xFAD mice, as well as improving the cognitive function of mice (Wang et al., 2024). Fluorescent imaging of AD human brain tissue showed that the expression of HSV-1 protein, ICP27, significantly increased toward more advanced stages of AD, and was co-localized with Iba-1 (microglial marker) and pTau in AD (Hyde et al., 2025). Co-localization of cGAS-STING proteins, such as NF-κB and IRF-3, and pTau at Ser396 was shown in the entorhinal cortex and hippocampus of AD tissue compared to control tissue, and the activation of cGAS-STING by the treatment of cGAMP in the absence or presence of TBK1 inhibitor in primary neuronal culture increased tau phosphorylation (Hyde et al., 2025). Hyde et al. proposed that tau phosphorylation is downstream of the cGAS-STING-TBK1 pathway, followed by HSV-1 infection (Hyde et al., 2025). The accumulation of damaged mitochondria is also one of the hallmarks of aging and AD, and mitophagy selectively removes damaged mitochondria (Youle and Narendra, 2011; Fang et al., 2019). Electron microscopy images showed that defective mitophagy is present in the hippocampus of postmortem human AD tissue (Fang et al., 2019). The induction of mitophagy by potent neuronal mitophagy-inducing agents such as urolithin A and actinonin improved microglial phagocytic activity for Aβ plaques and reduced inflammation in APP/PS1 transgenic AD mice, and reduced hyperphosphorylation of tau in 3xTg-AD transgenic AD mice (Fang et al., 2019). For HSV-1 infection, Oh et al. have shown that HSV-1 encoded protein kinase US3 inhibited PINK1/Parkin-mediated mitophagy, and HSV-1 infected microglia were associated with upregulated DAM-related gene expression patterns, including immunological response and inflammatory pathway genes (Oh et al., 2025). A mitophagy-inducing drug, ALT001, improved microglial phagocytic activity for uptaking oligomeric Aβ42 in microglial cell culture, followed by HSV-1 infection (Oh et al., 2025).

## 5 Discussion

In recent decades, a growing number of studies indicate that the potential pathological implication of infection is the risk of developing AD. However, one should be cautious in interpreting these association data, and it is worth noting that precise underlying mechanisms are still limited. For HSV-1, latent HSV-1 can be reactivated periodically in life (Marcocci et al., 2020; Wilson and Mohr, 2012). Thus, whether prevention and more longitudinal studies with therapeutic strategies to lower HSV-1 infection in general could slow or reduce the aggregation processes of Aβ and pTau, neuroinflammation, and the progression of AD would be worthwhile to investigate. At the same time, monitoring for HSV infection could bring a potential health benefit in individuals with a family history of AD (Liu et al., 2025). In addition, the effect of infection in combination with the effects of gender and/or genotypes such as one or two copies of the APOE4 allele could provide further insight into understanding the etiology of SAD, which is a complex mixture of genetic and environmental factors. Concurrently, further studies are needed to elucidate precise molecular mechanisms linking the viral-immune-brain axis. Therefore, those studies can demonstrate whether the link between viral infection and AD is causal or associative.
